# Short-wave enhances mesenchymal stem cell recruitment in fracture healing by increasing HIF-1 in callus

**DOI:** 10.1186/s13287-020-01888-0

**Published:** 2020-09-07

**Authors:** Dongmei Ye, Chen Chen, Qiwen Wang, Qi Zhang, Sha Li, Hongwei Liu

**Affiliations:** 1 Department of Rehabilitation, Affiliated Zhongshan Hospital of Dalian University, Dalian, 116001 China; 2 Department of Anatomy, Medical College of Dalian University, Dalian, China; 3 Department of Rehabilitation, The people’s Hospital of Longhua District, Shenzhen, China

**Keywords:** Mesenchymal stem cells, Short-Wave therapy, Recruitment, Fracture, HIF-1

## Abstract

**Background:**

As a type of high-frequency electrotherapy, a short-wave can promote the fracture healing process; yet, its underlying therapeutic mechanisms remain unclear.

**Purpose:**

To observe the effect of Short-Wave therapy on mesenchymal stem cell (MSC) homing and relative mechanisms associated with fracture healing.

**Materials and methods:**

For in vivo study, the effect of Short-Wave therapy to fracture healing was examined in a stabilized femur fracture model of 40 SD rats. Radiography was used to analyze the morphology and microarchitecture of the callus. Additionally, fluorescence assays were used to analyze the GFP-labeled MSC homing after treatment in 20 nude mice with a femoral fracture. For in vitro study, osteoblast from newborn rats simulated fracture site was first irradiated by the Short-Wave; siRNA targeting HIF-1 was used to investigate the role of HIF-1. Osteoblast culture medium was then collected as chemotaxis content of MSC, and the migration of MSC from rats was evaluated using wound healing assay and trans-well chamber test. The expression of HIF-1 and its related factors were quantified by q RT-PCR, ELISA, and Western blot.

**Results:**

Our in vivo experiment indicated that Short-Wave therapy could promote MSC migration, increase local and serum HIF-1 and SDF-1 levels, induce changes in callus formation, and improve callus microarchitecture and mechanical properties, thus speeding up the healing process of the fracture site. Moreover, the in vitro results further indicated that Short-Wave therapy upregulated HIF-1 and SDF-1 expression in osteoblast and its cultured medium, as well as the expression of CXCR-4, β-catenin, F-actin, and phosphorylation levels of FAK in MSC. On the other hand, the inhibition of HIF-1α was significantly restrained by the inhibition of HIF-1α in osteoblast, and it partially inhibited the migration of MSC.

**Conclusions:**

These results suggested that Short-Wave therapy could increase HIF-1 in callus, which is one of the crucial mechanisms of chemotaxis MSC homing in fracture healing.

## Introduction

Fracture healing is a biologically optimized process. Mesenchymal stem cells (MSCs) are multipotent stromal cells that can differentiate into multiple cell types such as chondrocytes and osteocytes and thus have an essential role in the bone healing process [1]. While fracture healing, circulating MSC can receive signals from the injured tissue and migrate to the damaged sites. Over the last decade, several strategies for fracture healing have been investigated, including the stimulation of endogenous stem cell populations from the mature body [2, 3]. An alternative strategy is the use of exogenous stem cells, which can be obtained from connective tissues and are controlled by the expression of molecules during MSC expansion, such as CXCR4 and complement 1q (C1q) [4, 5], or by certain chemicals [6], such as valproate or lithium, which are involved in MSC homing and can trigger the expression of certain key factors. Since the mobilization is a kind of directional migration, both endogenous and exogenous MSC recruitment is related to the condition of the fracture site. Currently, researchers are focusing on improving the condition of MSC recruitment by using biological agents. Clinical-standard platelet products loaded membranes [7] and naringin [8] successfully support MSC colony formation and promote MSC migration in vitro. Furthermore, MSC migration can be improved under hypoxic conditions. Previous studies have suggested that hypoxic conditions decrease the MMP secretion and increase CXCR4 expression [9, 10]. However, the effect of physical agency therapy on MSC migration has not received adequate attention.

Short-Wave therapy is a type of high-frequency electrotherapy, which can promote the fracture healing process [11, 12]. At high frequencies, the electromagnetic energy is converted to thermal energy, which can induce heat (temperature over 40 °C) to the treated area of the body [13], where the heating process affects blood flow [14] and decreases pain [15]. Accumulating evidence has indicated that both hypoxia and hypoxia-driven angiogenesis can be regulated by thermal therapy [16]. The hypoxia-inducible factor (HIF) involved signaling pathway is activated under hypoxia. HIF protein, especially HIF-1α, has been associated with MSC migration and differentiation [17]. As a critical transcriptional regulator, HIF-1 can regulate the expression of multiple cytokines, such as stromal cell-derived factor 1 (SDF-1) [10, 18] and focal adhesion kinase (FAK) [19], which has a central role in the adaptation of MSC to hypoxia [20, 21].

**Fig. 1:**
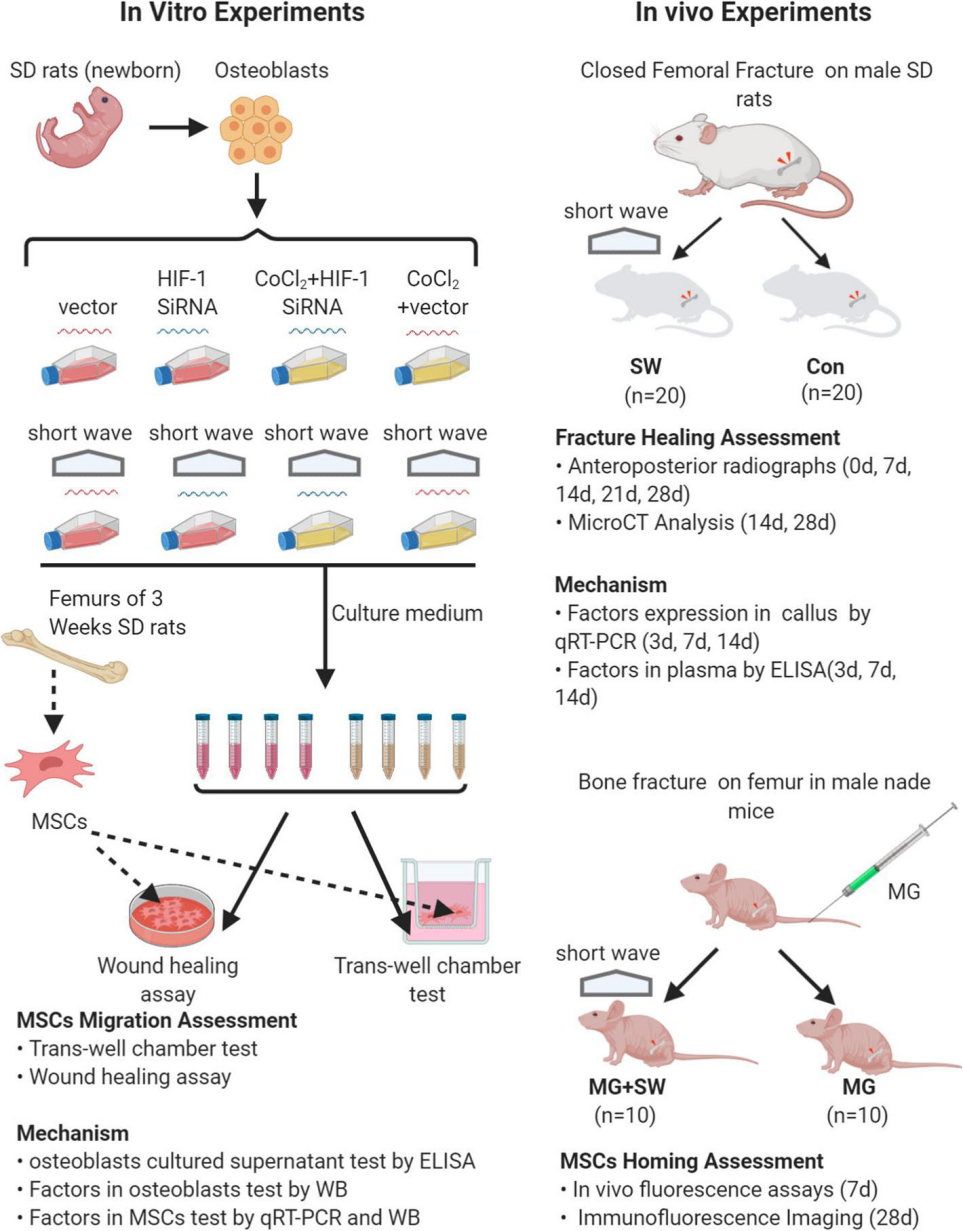
Flowchart of the study design. Stabilized femur fractures were established in 40 SD rats. Radiographs and micro-CT analysis examined the effect of Short-Wave on fracture healing. The expression of HIF-1 and other factors in callus was tested by q RT-PCR. SDF-1 in plasma was evaluated by ELISA. To analyze the MSC migration in healing, in vivo fluorescence assays and immunofluorescence were used after treatment in 20 nude mice with a femoral fracture. For in vitro study, osteoblast simulated fracture site was first irradiated by the Short-Wave; CoCl_2_ in medium stimulated hypoxia condition; siRNA targeting HIF-1 was used to investigate the role of HIF-1. Osteoblast culture medium was then collected as chemotaxis content of MSC, and the migration of MSC was evaluated using wound healing assay and trans-well chamber test. The expression of HIF-1 and its related factors were quantified by q RT-PCR, ELISA, and Western blot. SW Short-Wave treatment, MG GFP-labeled MSC. The image element in the flowchart mainly comes from https://app.biorender.com/

The present study explored the effects of Short-Wave therapy on HIF-1 expression in fracture and MSC recruitment from peripheral blood during fracture healing. Accordingly, we applied Short-Wave treatment on the fracture in the animal model and assessed the healing of fracture using the radiographs. Also, in vivo bioluminescent assays and callus histo-immunofluorescence were applied to evaluate the MSC migration. HIF-1 and related factors were also detected. siRNA targeted HIF-1α was transfected to clarify its effect in Short-Wave therapy (Fig. 1). This study aimed to investigate the impact of Short-Wave therapy on MSC recruitment and explore the underlying mechanisms of Short-Wave therapy on fracture healing.

## Materials and methods

### MSC isolation and identification

MSC was isolated from femurs of 3-week-old male SD rats, as previously described [22]. Before performing any experiment, cells were passaged for 3 to 6 times. The surface marker expressions of MSC, including CD90, CD44, CD34, CD45, and CD11b/c, were analyzed by flow cytometry assay, as described in the previous study [23, 24].

### Animal grouping

A total of 40 male SD rats, 8–12 weeks old, weighing 350–500 g, were obtained from Laboratory Animal Center of Dalian Medical University, China. Rats were housed in an environment with a temperature of 22 ± 1 °C, a relative humidity of 50 ± 1%, and a light/dark cycle of 12/12 h.

After 1 week of adaptation, 40 SD rats were accurately weighed and randomly divided into two groups (*n* = 20/group): Short-Wave treatment group (SW) and control group (Con). All rats underwent surgery to establish the femoral shaft fracture and intramedullary fixation model.

For in vivo bioluminescent assays to the MSC homing, 20 nude mice of femoral shaft fracture were injected MSC labeled by the GFP (MG) and then randomly divided into two groups, including a Short-Wave treatment group (MG+SW) and a control group (MG) (*n* = 10/group).

### Stabilized fracture model

Stabilized right femur fractures were established by intramedullary fixation in 8–12-week-old male SD rats. A 0.25-mm titanium alloy pin was inserted inside the medullar canal of the femur. A three-point bending device was then used to produce closed fractures of the femoral shaft with a standardized force [25]. In 20 male nude mice, transverse osteotomy with a 1-mm bone fracture was created in the middle of the right femur in the same way. Buprenorphine was subcutaneously administered (0.5 mg/kg) for pain control.

### Cell injection

All the 20 nude mice received injections with 5 × 10^6^/50 μl GFP-labeled MSC (MG) (C57BL/6 mouse strain) (CYAGEN Company, China) 3 days after the femoral fracture. Cells were injected by tail vein using a microinjector.

### Short-Wave treatment

Three days after the operation, Short-Wave therapy was applied in the Short-Wave treatment group. Briefly, animals were fixed in an apparatus so that they could not turn around. The treatment regimen was applied to the right thigh. The Short-Wave generator operated at a frequency of 27.12 MHz. A micro-heat continuous-wave Short-Wave exposure for 10 min was applied once a day. A similar procedure was carried out in the control group; however, the device was turned off.

For the in vitro study, the isolated rat osteoblasts in the treatment group underwent Short-Wave irradiation. The protocol of Short-Wave therapy irradiation on cells was based on a previous research [26]. The two non-contact applicators were of perpendicular contraposition and 10 cm away from each other. The cell culture flask was placed in the middle of the applicators. A micro-heat continuous-wave Short-Wave exposure for 90 min was applied twice on day 1 in the open air at 37 °C. The control group received a sham Short-Wave treatment by turning off the Short-Wave generator. On day 2, the culture medium from each group was collected for HIF-1 and SDF-1 protein-level analysis, and the medium was used in MSC cell wound healing assay and trans-well chamber test.

### Radiographic assessment

The fracture healing was assessed by plain anteroposterior radiographs at days 7, 14, 21, and 28. The same X-ray machine and settings were used for all radiographs every 7 days after fracture. Bone defects were analyzed using X-ray software.

Micro-CT scanning was performed to assess callus. Quantification for the volumes of the bony calluses as determined as previously described [27, 28]. The region of interest was set within 800 μm (50 slices) around the defect edge. We applied a fixed threshold of less than 330 for new calcified cartilage or unmineralized cartilage. Three-D microstructural image data were reconstructed by Inveon Research Workplace software. After 3D reconstruction, bone volume fraction (BV/TV) was automatically determined to confirm fracture healing.

### In vivo fluorescence assays

Ten nude mice per group received anesthesia and were examined by the IVIS imaging system at day 7 after the surgery. The identical parameter settings were used for all samples: *f* number 1, field of view 22, binning factor 18, and luminescent exposure (seconds) 10. The IVIS imaging examination and rates of photons were calculated and performed according to methods reported in a previous study [29].

### Histological analysis and immunofluorescence imaging

Twenty nude mice (MG: *n* = 10, MG + SW: *n* = 10) were sacrificed for histological analysis and immunofluorescence imaging at day 28 after the operation. The femoral bone of nude mice and rats were sectioned, preserved, decalcified, and embedded in paraffin along the longitudinal axis. For morphological analysis, 5-μm slices were sectioned, deparaffinized, and stained using hematoxylin and eosin. The immunofluorescence staining was performed as previously described [30]. Tissue slides of nude mice were stained with antibodies (A0516, Beyotime, China) against GFP to track exogenously delivered MSC labeled by GFP. A four-channel confocal laser scanning microscope was used to analyze all the samples. GFP-positive cells were automatically counted in five fields on × 100 magnification by ImageJ software.

### Enzyme-linked immunosorbent assay (ELISA)

The plasma of SD rats received by heart puncture at days 3, 7, and 14 after the operation (*n* = 4/group/time point). The concentration SDF-1 in plasma of SD rats was analyzed using citrulline ELISA kit (CSB-E13414r, Cusabio Biotech, China). In culture media of osteoblasts (SD rat strain), the concentration of HIF-1 (SEA798Ra, USCN, China) and SDF-1 (SEA122Ra, USCN, China) content was also detected using the ELISA kit.

### Quantitative reverse transcription-polymerase chain reaction (q RT-PCR)

Callus of sacrificed SD rats was collected and snap-frozen in liquid nitrogen at days 3, 7, and 14 after the operation (*n* = 4/group/time point). RNA isolation and subsequent cDNA synthesis (Bio-Rad, 170-8891) were performed as previously described [31]. A total of 50 ng of cDNA was amplified with custom-designed q RT-PCR primers (Table 1). A melt curve was generated to analyze the purity of amplification products. The expression levels of mRNA were normalized to the average of β-actin. Relative expression of mRNA was evaluated by using the comparative CT method (ΔΔCt) [32].

**Table 1:** Oligonucleotide product size and accession numbers for q RT-PCR

Gene	Product size	Accession number
HIF-1	105	NM_021704
SFD-1	126	NM_001033
FAK	130	NM_013081
F-actin	127	NM_001109
CXCR4	116	NM_009911
β-catenin	155	NM_ 053357
β-actin	103	NM_007393

### Osteoblast culture and identification

Osteoblasts were obtained from calvaria of 1-day-old neonatal SD rats using the method of collagenase-pancreatic enzyme digestion as detailed in reference [33]. After two passages, alkaline phosphatase staining was utilized to identify the osteoblast cells.

### Small interference RNA transfection

We inhibited HIF-1α expression by siRNA in osteoblasts (SD rat strain). Synthetic siRNA oligonucleotide specific for HIF-1α (NM_024359) (5′ to 3′: UUUAUCAAGAUGGGAGCUCTT) and nontargeting siRNA were obtained from Sangon (Shanghai, China).

### Osteoblast culture medium

Osteoblasts (SD rat strain) were seeded in 6-well plates for 24 h to 80–90% confluence. Three kinds of interventions were provided: (1) Short-Wave continuous irradiation for 180 min, (2) 200 μmol/l CoCl_2_-stimulated hypoxia condition in fracture, and (3) siRNA inhibition of HIF-1α. Eight kinds of osteoblast culture mediums were obtained from single or combined interventions for the follow-up experiment (Fig. 1).

### Wound healing assay

MSC (SD rat strain) was cultured on 6-well plates to confluency and monolayers and wounded with a sterile 200-μl pipette tip. The cultures were washed with PBS to remove detached cells and stimulated with 8 kinds of osteoblast culture medium fluid 1.5 ml in each well. Photographs were collected at 0, 24, and 48 h.

### Trans-well chamber test

The tests were performed in Boyden Chambers (Corning, 3422, Lowell, MA). Eight kinds of osteoblast (SD rat strain) culture medium were seeded in the bottom chamber. The top chambers filled with MSC (SD rat strain) starved overnight were inserted. Twenty-four hours later, inserts were removed and washed. The cells that migrated to the bottom side were accumulated.

### Western blotting

Osteoblasts and MSC cell (both are SD rat strain) lysates were prepared, and western blots were performed as previously described [34]. The 30 μg of protein was loaded in each lane for reducing electrophoresis. Primary antibodies were used for β-actin (Sigma; A2228) diluted 1:10000, HIF-1α (Gene Tex; GTX127309) diluted 1:2000, SDF-1 (CST; 3740) diluted 1:1000, FAK (Sangon Biotech; D160324) diluted 1:3000, phosphor-FAK (Sangon Biotech; D160324) diluted 1:2000, F-actin (Abcam; Ab205) diluted 1:2000, β-catenin (wanleibio; WL0962a) diluted 1:500, and CXCR4 (Abcam; ab124824) diluted 1:1000.

### Statistical analysis

Statistical analysis was performed by SPSS 22.0 for Windows (SPSS, Chicago, USA). The results are shown as the mean value ± standard deviation. The differences between groups were analyzed by *t* test or analysis of variance (ANOVA). Two-tailed *P* values were computed, and *P* < 0.05 was considered to be statistically significant.

## Results

### MSC identification

**Fig. 2:**
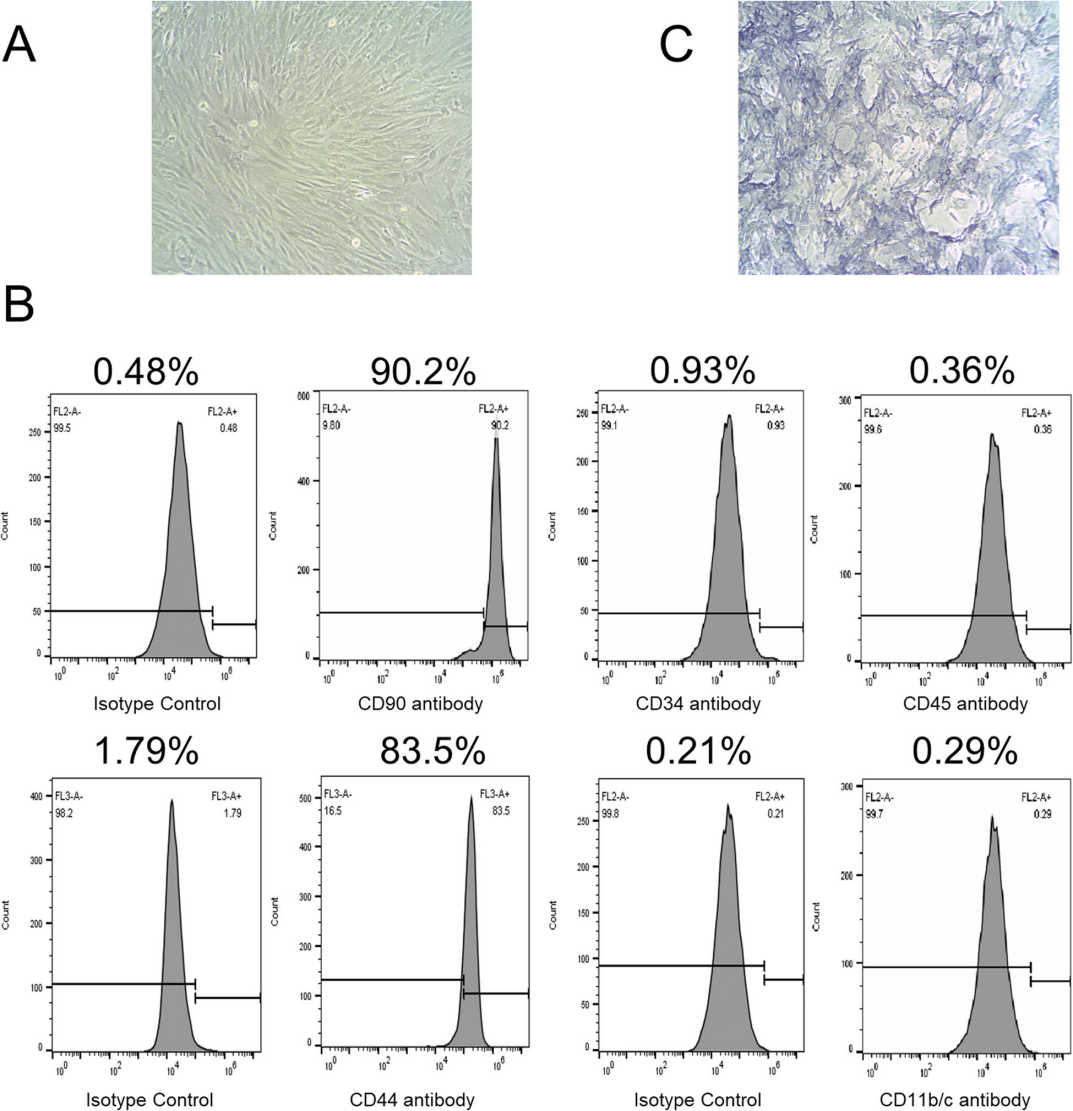
MSC cells used in this study. **a** MSC from SD rat had a fusiform shape and was arranged in bundles or whorls. **b** MSC established from cells in the primary culture was stained for high-affinity receptors CD90 and CD44 and the low-affinity receptor CD34, CD45, CD11b/c with specific antibodies, and were then analyzed using flow cytometry. **c** The alkaline phosphatase staining method was used to identify primary cultured osteoblasts

After 3 to 6 passages, MSCs extracted from SD rats were in fusiform shape, arranged in bundles or whorls (Fig. 2a). The results of flow cytometry demonstrated that over 90.2% and 83.5% of the mononucleated cell colonies isolated from the bone marrow of SD rats were positive for fibroblastic marker CD90 and MSC marker CD44, respectively, and negative for hematopoietic lineage markers (CD45 and CD11b/c) and the endotheliocyte lineage marker CD34 (0.36%, 0.29%, and 0.93%, respectively) (Fig. 2b).

### Short-Wave treatment enhancing fracture healing

**Fig. 3:**
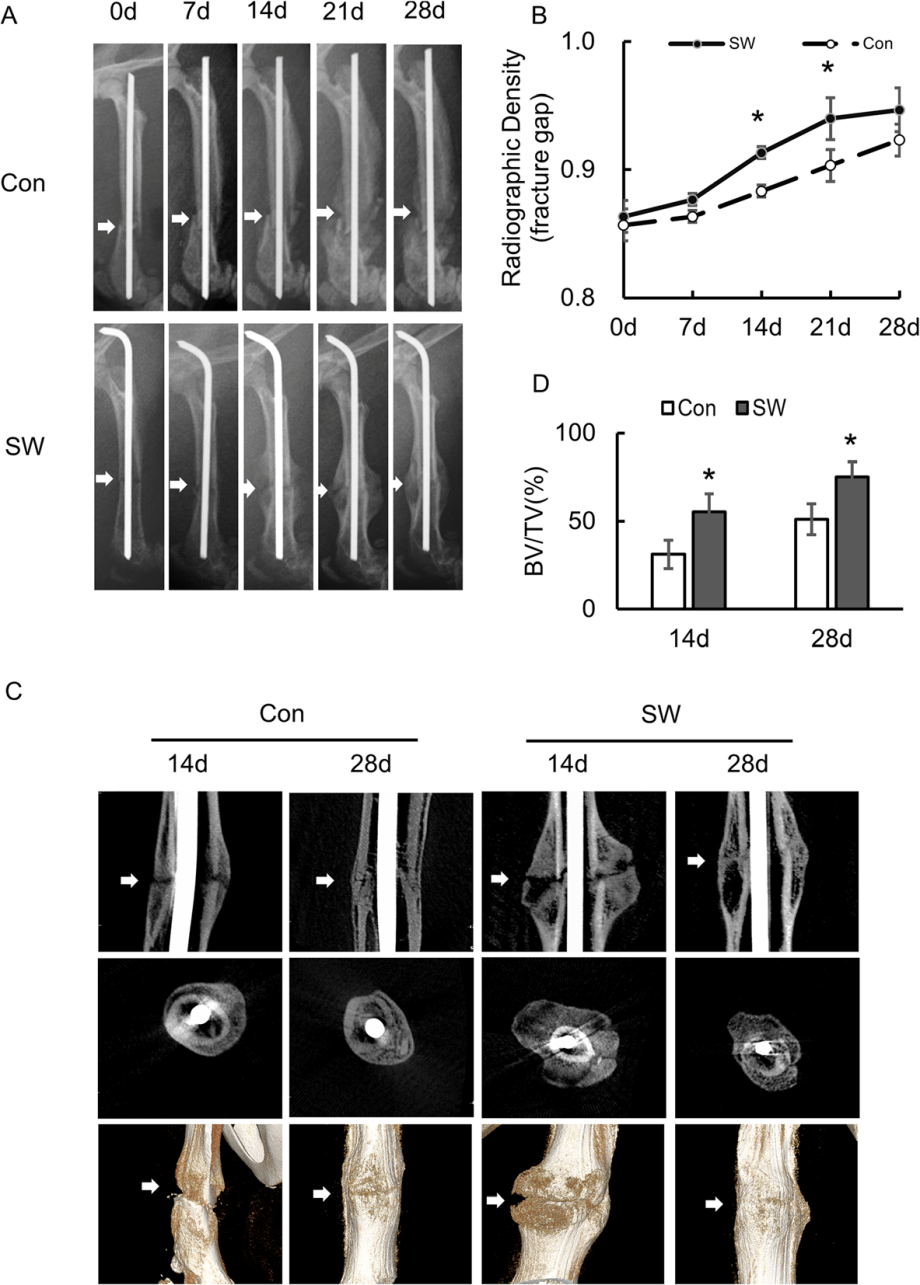
Radiographs and micro-CT of the femur. **a** Radiographs of SD rat’s femur. Callus formation was seen in the control group as well as in the SW group (*n* = 4/group). **b** The normalized radiographic density of the femur in control and different treatment. The higher radiographic density turned out in the SW group at day 14 and day 21 post-operation. After 28 days, no obvious fracture gap was found in each group. **c** Micro-CT images of the fracture (SD rats). More callus and narrow gaps were seen in the SW group at day14 (*n* = 4/group). **d** Analysis of ROI bone volume fraction (BV/TV) in the control and SW groups. Arrows in **a** and **c** represent fractures. Data represent mean ± SD. Differences were assessed on each day by performing one-way ANOVA. *P < 0.05. Con control, SW Short-Wave treatment

To examine the role of Short-Wave treatment and MSC during fracture repair, we generated a closed femoral shaft fracture model. Radiographic examinations showed a normal bone healing process in all rats. The radiographic analysis demonstrated that the SW group showed better fracture healing than the control group (Fig. 3a). Besides, the quantitative measurement density of the fracture gap showed that SW groups were significantly larger than the control group at days 14 (*P* = 0.033) and 21 (*P* = 0.026). Nevertheless, no significant difference was found between days 7 (*P* = 0.152) and 28 (*P* = 0.163) (Fig. 3b).

Micro-CT analysis showed that a more significant amount of bony callus was found in the SW groups than in the control group. Three-D reconstructed micro-CT images on day 14 and 28 post-fracture are shown in Fig. 3c. Comparing with the control group, the bone volume fraction (BV/TV) in the SW group was significantly higher at day 14 (*P* = 0.045) and day 28 (*P* = 0.049) (Fig. 3d).

### Short-Wave therapy treatment promoting MSC homing in vivo

**Fig. 4:**
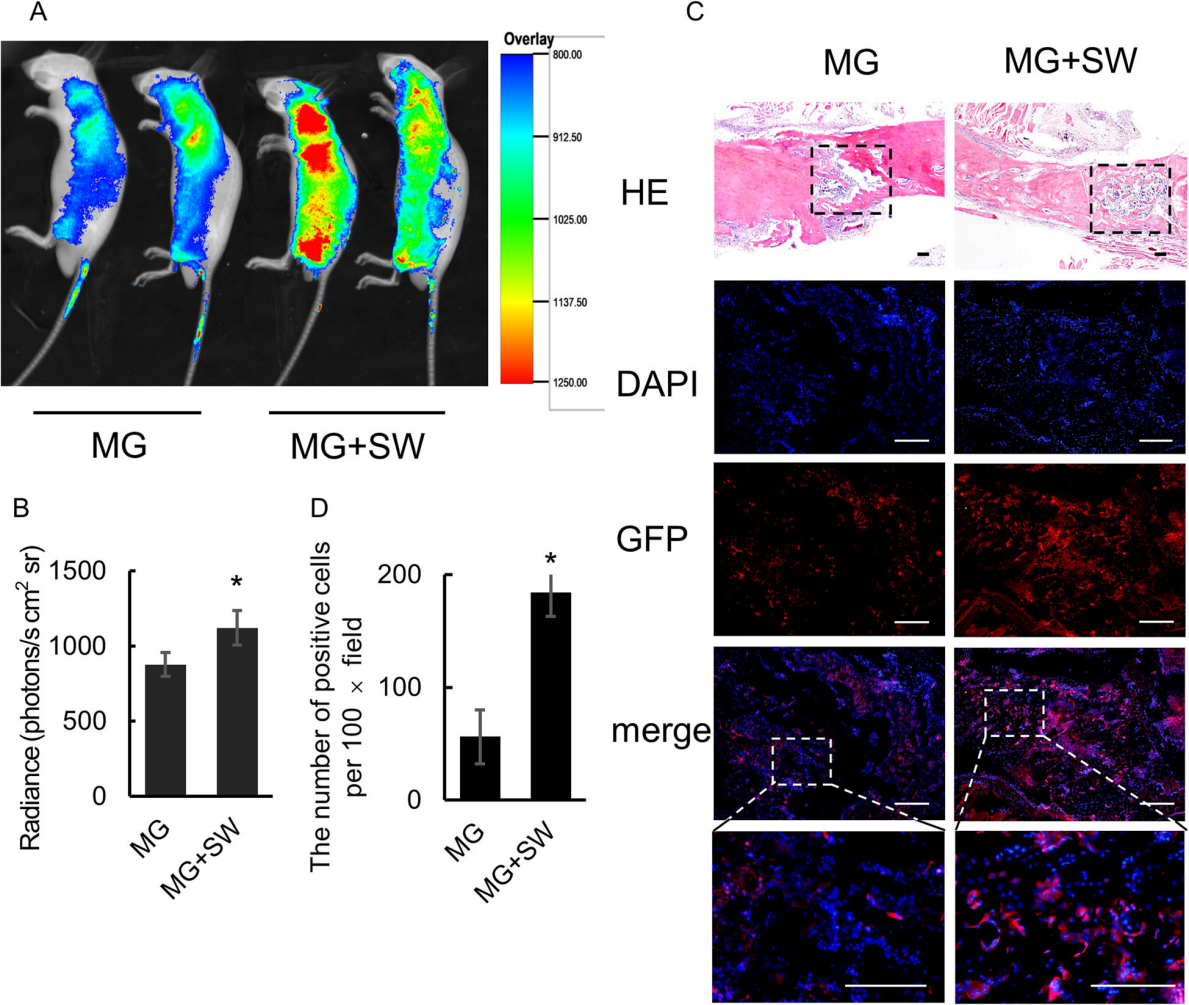
Tracing MSC homing in nude mice. **a** Fluorescence assays in vivo. Nude mice of femoral shaft fracture were injected MSC labeled by the GFP (MG) and then randomly divided into two groups, including a Short-Wave treatment group (MG+SW) and a control group (MG) (*n* = 10/group). MG was examined by an IVIS imaging system 7 days after the operation. **b** Analysis of total radiant efficiency in the right femur. **c** Homing of MG to the fracture site, analyzed by histological analysis and immunofluorescence imaging at 28 days after the operation. The rectangular boxes in HE staining sections represent fracture areas, which is the region of interest in immunofluorescence imaging. The MG shown in red accounted for Cy3-labeled antibody. Scale bars 200 μm. **d** Six high-magnification fields (× 100) were randomly observed, and the number of GFP-positive cells was counted and analyzed. Significantly more MG cells were counted in the femora of animals treated with Short-Wave irradiation. Data represent mean ± SD. Differences were assessed by performing a *t* test. *P < 0.05. MG injected MSC labeling with the GFP, SW Short-Wave treatment

Nude mice were injected with MSC expressing the GFP report gene for observation of the MSC homing to the fracture. IVIS imaging system recorded radiant efficiency in the leg at day 7 post-operation. Higher cell migration was more common in animals receiving Short-Wave irradiation with total radiant efficiency [p/s]/[μW/cm^2^] compared to those not exposed to wave irradiation (1123.1 ± 116.0 vs 878.2 ± 79.2, *P* = 0.023; Fig. 4a, b). Besides, GFP distribution analysis showed that MSC was not uniformly distributed throughout the body and tended to migrate to organs such as the lung, liver, and tail, which was observed in both groups.

Next, we quantified the homing of exogenously delivered MSC to the fracture site by using immunofluorescent staining and histological analysis. All the nude mice were sacrificed, and the callus tissues were prepared for immunofluorescence staining at day 28 after the operation. Significantly more MG cells were counted in the femora of animals treated with Short-Wave irradiation (GFP-positive cells per × 100 field 56.3 ± 26.8 vs 184.3 ± 21.2, *P* = 0.003; Fig. 4c, d).

### Short-Wave therapy changes the gene expression in callus

**Fig. 5:**
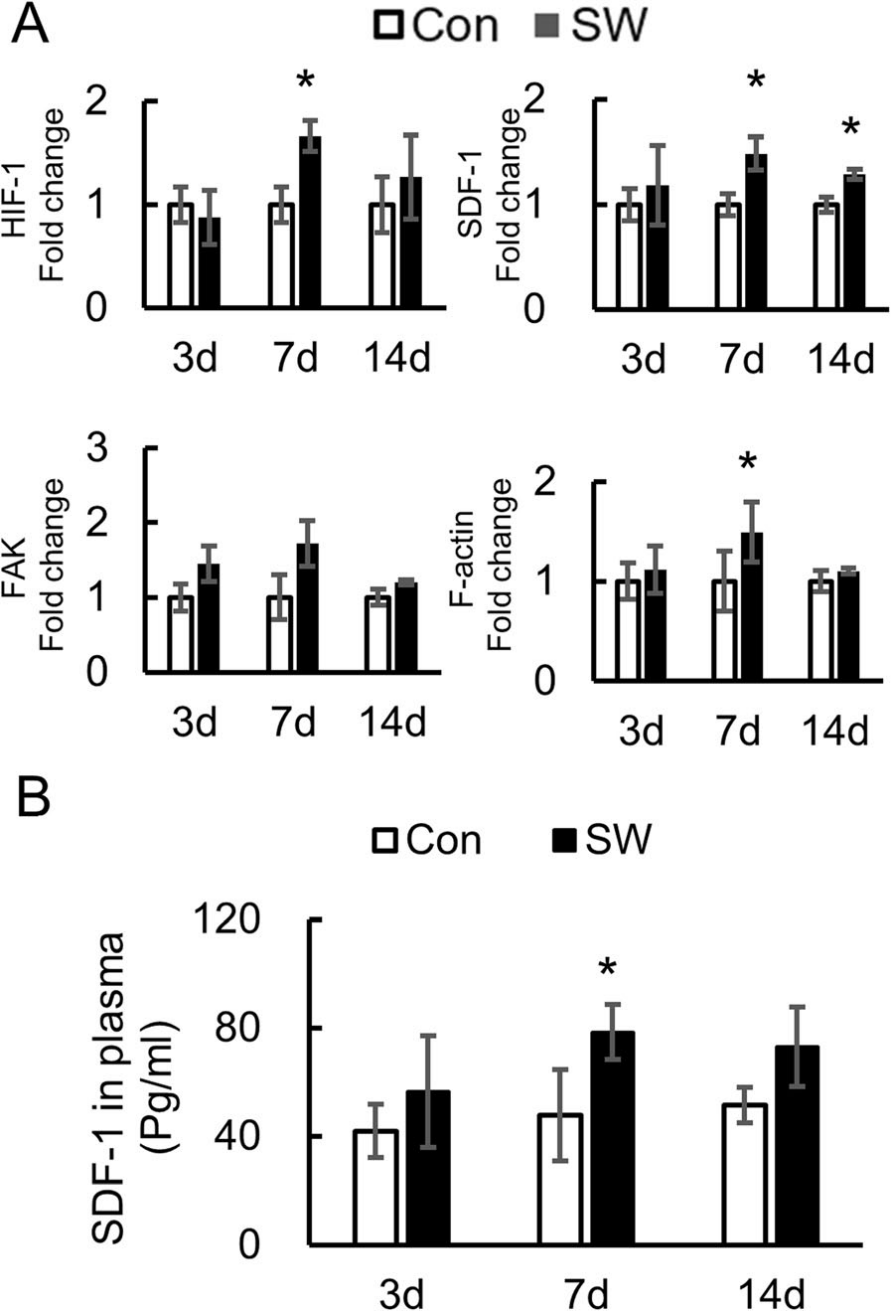
Factors’ expression in vivo. **a** Callus of sacrificed SD rats was collected at days 3, 7, and 14. The expression of HIF-1, SDF-1, FAK, and F-actin in callus tissue detected by q RT-PCR (*n* = 4/group/time point). **b** The concentration of SDF-1 in plasma of sacrificed SD rats detected by ELISA at days 3, 7, and 14 (*n* = 4/group/time point). Data represent mean ± SD. Differences were assessed among the four groups on each day by performing one-way ANOVA. *P < 0.05. Con control, SW Short-Wave treatment

The results above indicated that Short-Wave therapy enhanced MSC migration to the fracture. Low oxygen occurs in the fracture following bony injury [35]. Therefore, the involvement of HIF-1 and its related factors (SDF-1, F-actin, and FAK) were investigated after Short-Wave therapy using q RT-PCR. No significant differences in HIF-1 were detected between the two groups at day 3 (*P* = 0.706) and 14 (*P* = 0.602) post-therapy, yet its expression significantly increased in the SW group at day 7 compared to the control group (*P* = 0.002; Fig. 5a). Moreover, compared with the control group, the expression of SDF-1 increased in the SW group on days 7 (*P* = 0.044) and 14 (*P* = 0.016). In addition, a significant increase of F-actin was found at day 7 in the SW group (*P* = 0.038), while there was no difference at day 3 (*P* = 0.728) and day 14 (*P* = 0.835). For FAK, it seemed that Short-Wave therapy led to increases on days 3 and 7; nevertheless, there was no statistical difference between the two groups at day 3 (*P* = 0.051), day 7 (*P* = 0.142), and day 14 (*P* = 0.287).

In addition, SDF-1 in plasma was detected by ELISA (Fig. 5b). After 7 days of treatment with Short-Wave therapy, SDF-1 was increased by 1.5-folds compared to the control group (*P* = 0.044).

### Short-Wave therapy promotes HIF-1 expression in osteoblasts in vitro

**Fig. 6:**
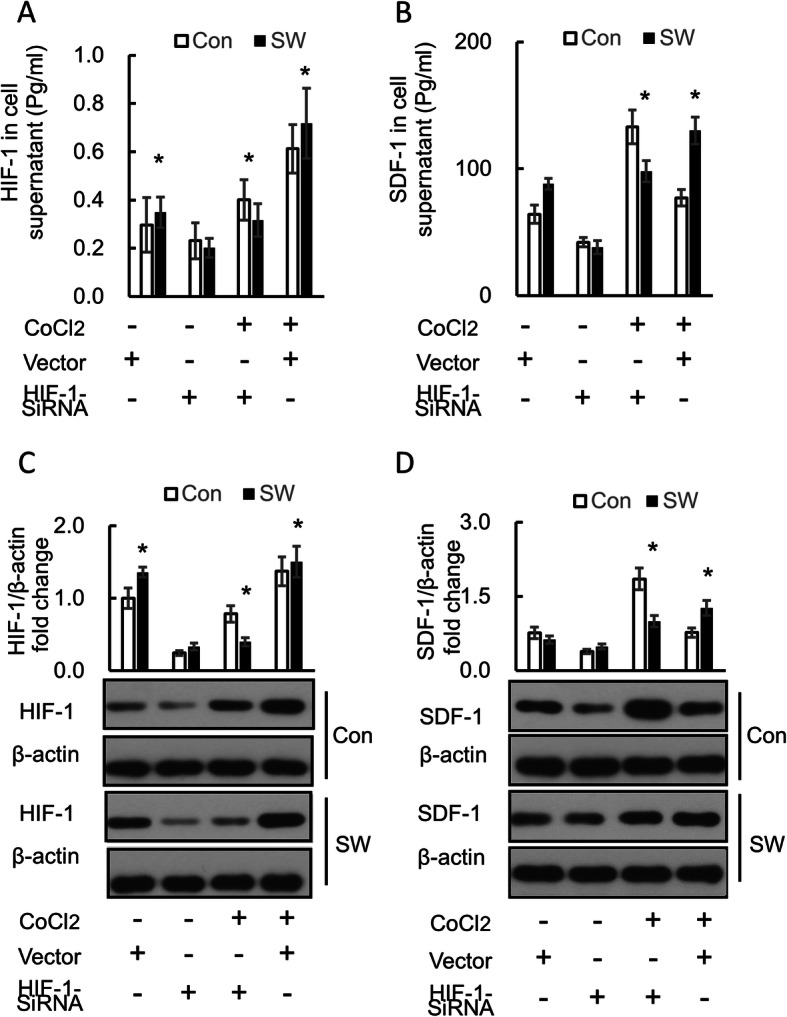
Factors’ expression in osteoblasts in vitro. Osteoblasts of SD rats were fed in four kinds of medium. Short-Wave irradiation was provided to the cells in the SW group. **a**, **b** The concentration of HIF-1 and SDF-1 in the culture medium of osteoblasts was analyzed by ELISA. **c**, **d** The expression of HIF-1 and SDF-1 in the osteoblasts was analyzed by western blot. Data represent mean ± SD. Differences between the control group and the Short-Wave treatment group were assessed by performing a *t* test. *P < 0.05. Con control, SW Short-Wave treatment

We evaluated the HIF-1 in the culture medium fluid of osteoblasts under a Short-Wave in vitro by ELISA. The results showed that the expression of HIF-1 significantly increased following Short-Wave treatment (*P* = 0.047), especially under CoCl_2_ stimulated hypoxic conditions (*P* = 0.018). Next, we collected the osteoblasts and measured the expression of HIF-1 by western blot. We found a higher expression of HIF-1 after Short-Wave irradiation, both under normoxia (*P* = 0.021) and hypoxic condition (*P* = 0.046) (Fig. 6a, c). Also, the expression of SDF-1 in the medium improved following Short-Wave therapy under normoxia (Fig. 6b, d). HIF-1α-siRNA inhabited the HIF-1 expression. The expression of SDF-1 in osteoblasts and its concentration in culture media of osteoblasts was decreased after treating cells with HIF-1α-siRNA.

### The migratory effect of HIF-1 on MSC under Short-Wave irradiation

**Fig. 7:**
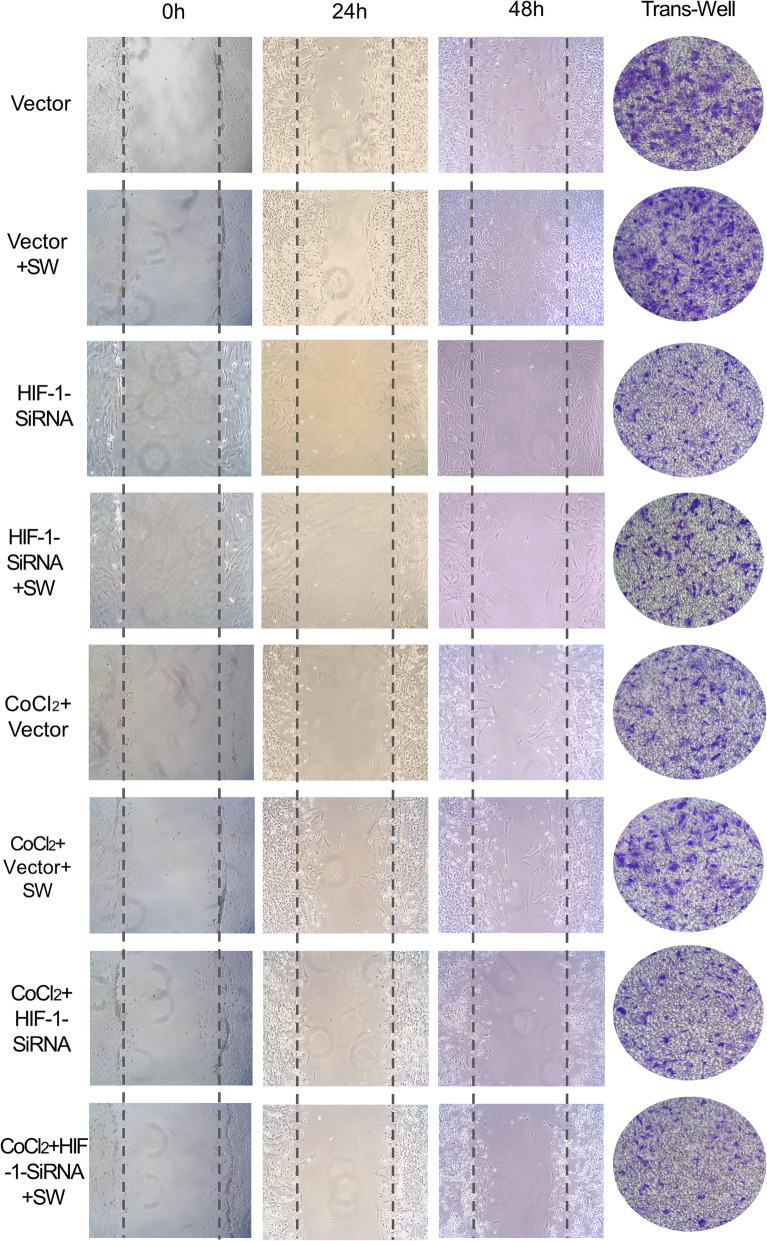
Migration in vitro. In wound healing assay, MSC (SD rat strain) was fed with 8 different kinds of osteoblast culture medium. Photographs were collected at 0, 24, and 48 h. Additionally, the chemotactic effect of the culture medium of Short-Wave-irradiated osteoblasts on MSC in vitro analyzed by the trans-well assay. Eight kinds of osteoblast (SD rat strain) culture medium were seeded in the bottom chamber. The top chambers filled with MSC (SD rat strain) starved overnight were inserted. Twenty-four hours later, the cells that migrated to the bottom side were accumulated

As shown in Fig. 7, MSC cultured in normoxia with the medium fluid of SW-irradiated osteoblasts infected vector for 24 h. At 48 h, it showed higher migration compared to control cells cultured in the medium fluid without SW irradiation (*P* < 0.05). The same tendency was also seen in simulated hypoxic conditions at 24 h (*P* < 0.05), but not at 48 h. The results of the analysis are seen in Table 2.

**Table 2:** Migration test in vitro

	Wound region (%WR)^a^		Number of migrated cells 24 h
	24 h	48 h	
Vector	31.4 ± 1.1	19.8 ± 0.8	319 ± 13
Vector+SW	27.7 ± 1.5^b^	8.84 ± 1.3^b^	420 ± 15^b^
Hif-1-SiRNA	44.9 ± 1.7	38.7 ± 3.7	221 ± 11
Hif-1-SiRNA+SW	46.1 ± 1.4	40.0 ± 1.5	229 ± 12
CoCl_2_+Vector	45.6 ± 0.9	32.2 ± 3.0	244 ± 10
CoCl_2_+Vector+SW	35.4 ± 2.2^c^	33.8 ± 3.4	374 ± 17^c^
CoCl_2_+Hif-1-SiRNA	45.9 ± 1.2	42.6 ± 1.7	221 ± 11
CoCl_2_+Hif-1-siRNA+SW	43.7 ± 1.6	38.8 ± 1.8	207 ± 13

^a^%WR = (the size of the wound region/total size of image) × 100. ^b^Vector VS Vector+SW at the same time point *P* < 0.05. ^c^CoCl_2_+Vector VS CoCl_2_+Vector+SW at the same time point *P* < 0.05

Additionally, to determine the effects of the HIF-1 in Short-Wave treatment on MSC migration, we used siRNA to inhibit HIF-1 in cultured osteoblasts. Briefly, siRNA reversed the above outcome (Fig. 7, Table 2).

### Migration-correlation factor expression in MSC

**Fig. 8:**
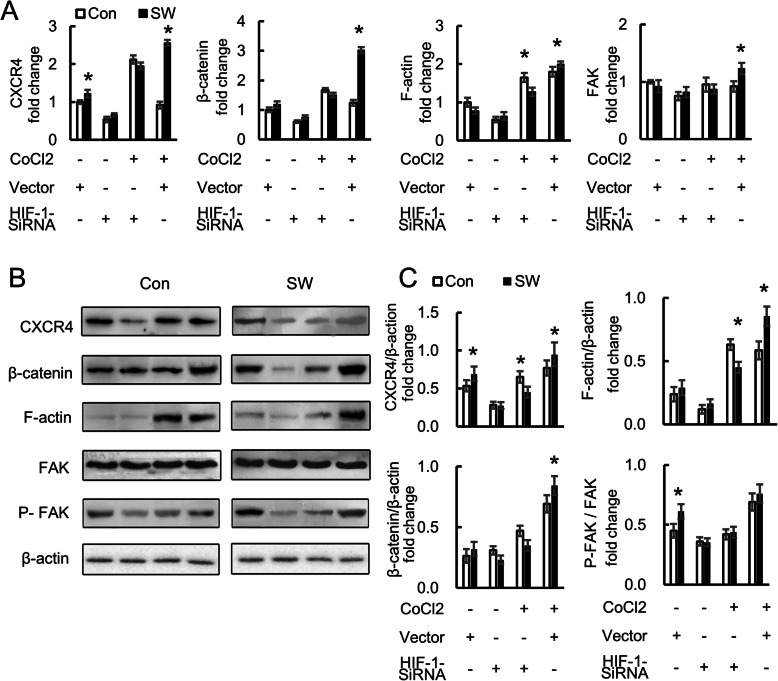
Factors’ expression in MSC. **a** MSC (SD rat strain) was fed with different kinds of osteoblast culture medium. The gene expression of CXCR4, β-catenin, FAK, and F-actin in MSC detected by q RT-PCR. **b** The presence of the factors as the protein of MSC was detected by western blot. **c** Quantification of protein bands from western blot films. The quantification will reflect the relative amounts as a ratio of each protein band relative to the lane’s loading control β-actin. The data of western blot represent mean ± SD. Differences between the control group and SW group were assessed by performing a *t* test. *P < 0.05. Con control, SW Short-Wave treatment

Next, we examined whether the medium fluid of osteoblasts irradiated by Short-Wave could affect the gene expression of con-cultured MSC. The gene expression of CXCR4, β-catenin, FAK, and F-actin in MSC were analyzed using q RT-PCR (Fig. 8a) and western blot (Fig. 8b, c). As a receptor, CXCR4 was increased under normoxia (*P* = 0.011) and under hypoxic conditions (*P* = 0.015). In addition, it decreased under HIF-1-inhabited medium both under normoxia (*P* = 0.900) and hypoxia (*P* = 0.046). The Short-Wave-irradiation medium also provided a positive effect on the expression of β-catenin (*P* = 0.013) and F-actin (*P* = 0.031) in MSC under hypoxia; yet, no statistical difference was observed under normoxia (each *P* > 0.05). The rise of F-actin and β-catenin were restrained since osteoblasts were transplanted in HIF-1 siRNA under normoxia and hypoxia (each *P* > 0.05). Additionally, no statistical difference was observed in FAK between the two groups (each *P* > 0.05). Nevertheless, phosphorylation levels of FAK were much higher in the Short-Wave group under normoxia (*P* = 0.040), which also decreased since MSC cultured in the medium of HIF-1 restrained osteoblasts (each *P* > 0.05).

## Discussion

The clinic application of high-frequency treatment can accelerate the resolution of hematoma and fracture healing. Nevertheless, the underlying mechanisms are not fully understood. Previously, it has been reported that fracture healing is associated with an increase in calcium phosphate mineral salt deposition, which occurs 2 to 4 weeks after injury [36, 37]. In this study, we found that high-frequency Short-Wave irradiation could promote the healing process, including the promotion of bony callus formation and the MSC migration. These healing characteristics were observed 1 to 3 weeks after the injury.

MSC has a vital role in the process of fracture healing [38]. The early stage of healing is mainly mediated by the biological actions of MSC [38]. Preclinical studies have suggested that transplanted exogenous or endogenous MSC can migrate to the fracture site after injury and restore the fracture callus volume and biomechanical properties of the bone in mice [39]. Injection of MSC by intravenous or intra-arterial infusion is commonly used to treat bone injury in mice. Yet, systemic administration has low therapeutic efficacy because only a small percentage of MSC can reach the target tissue [40, 41]. Hence, one of the solutions to improve therapeutic efficacy is to promote MSC migration and homing.

So far, only a few studies have focused on investigating the MSC migration in the electric field, especially in the direct current electric field. Zimolag and Griffin have observed that in an external direct current electric field, MSC directly migrated toward the cathode [42, 43]. Moreover, Banks et al. have discovered that cells display a highly elongated phenotype conversion and consistent perpendicular alignment to the electric field vector accounting for the effects of electric field strength [44]. Furthermore, Liu and Zhao found that the applied electromagnetic field might be useful to control or enhance the migration of MSC during bone healing [45, 46]. Nevertheless, as a kind of high-frequency electrotherapy, areas of exposure could not be polarized by Short-Wave therapy, the migration of MSC might not be associated with field stress. Short-Wave therapy is generally used for thermal effects that provoke or enhance cellular activity resulting from energy-absorbing in oscillating electrical fields [47]. Previous studies have shown that thermal stress can modulate some molecules affected by hypoxia [48, 49]. For example, HIF-1α, a transcription factor that is positively correlated with the acute temperature changes in organs, such as the brain, liver, kidney, and gonad tissues, can regulate the cellular response to hypoxia stress [50] that is significantly increased in osteoblasts [51]. In the current study, we did not focus on the direct effect of Short-Wave on the migration of MSC in vitro. We found an increased expression of HIF-1 in callus and osteoblasts exposed to Short-Wave therapy, which could be the chemokines for MSC to fracture area. Consequently, we assume that the regulation of the hypoxia pathway in callus affects MSC homing to promote tissue regeneration by Short-Wave. High expression of HIF-1a and BMP-2 promote the migration of MSC to the bone defect area [3]. Therefore, in several tissue engineering strategies, HIF stabilized biological materials are used to improve MSC migration and survival [52, 53]. In this study, it was found that exogenous MSC homing increased in fractures exposed to Short-Wave irradiation in vivo. Besides, we discovered that the MSC migration was improved by the cultural medium of osteoblast exposed to Short-Wave therapy irradiation in vitro, which could be further enhanced in stimulated hypoxia in fracture and restrained by inhibiting HIF-1α expression of culture osteoblasts. Therefore, we believe that HIF-1 is a key factor in the healing process activated by Short-Wave treatment. However, the effect of Short-Wave treatment on fracture healing of the HIF-1a inhibitor was not observed in vivo, which is a limitation of the current study. A research of HIF-1a conditional knockout rats of fracture with Short-Wave therapy will be conceived in the future.

**Fig. 9:**
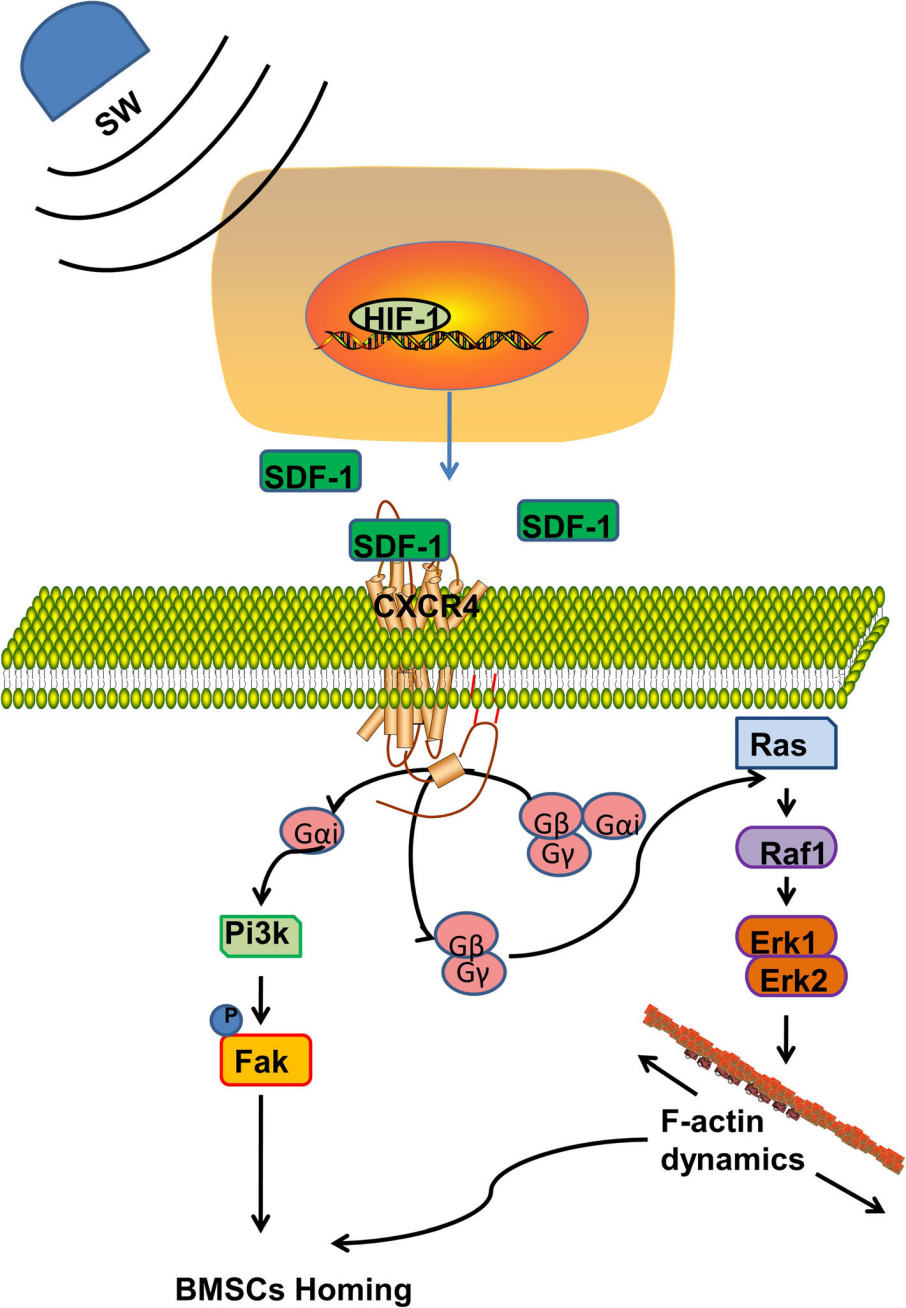
Schematic diagram of Short-Waves promoting fracture healing by chasing HIF-1 and promoting MSC migration. SW Short-Wave treatment, HIF-1 hypoxia-inducible factor 1, SDF-1 stromal cell-derived factor 1, CXCR4 C-X-C motif receptor 4, PI3K phosphatidylinositol 3-kinase, Erk extracellular regulated protein kinases, FAK focal adhesion kinase, MSCs mesenchymal stem cells

The homing of CXCR4-positive progenitor cells in circulation is upregulated since the increase of HIF-1 induces SDF-1 expression. As a cell growth-stimulating factor, SDF-1 belongs to the CXC subfamily of chemokines [54, 55]. SDF-1 can activate CXCR4, a G protein-coupled receptor [56]. Progenitor cell recruitment to injured tissues can be prevented if SDF-1 in ischemic tissue or CXCR4 on circulating cells were blockaded [54, 57]. In the bone marrow, discrete regions of the anoxic chamber have increased SDF-1 expression and progenitor cell tropism [58]. Over the last 10 years, numerous studies have confirmed that SDF-1/CXCR4 has a pivotal role in the biologic and physiologic functions of MSC [59]. In this study, we found increased expression of SDF-1 in callus and blood exposed to irradiation, which serves as a chemoattractant to recruit CXCR4-expressed MSC both in circulation and fracture site. FAK is the downstream protein kinase in the CXCR4 signaling cascade, which can integrate extracellular signaling and cellular migration [60]. Additionally, the cytoskeleton network partly detects the biomechanical characterization of living cells. It has been suggested that F-actin transmutation affects cell morphology and migration [61]. In the current study, the expression of F-actin and phosphorylated FAK increased in the medium of cultured osteoblasts under Short-Wave irradiation. The migration of MSC was improved on the molecular level. Therefore, Short-Wave treatment enhanced the local chemotaxis for MSC in the fracture site, which might be the underlying mechanism (Fig. 9).

## Conclusion

Short-Wave therapy could increase HIF-1 in callus, which is one of the crucial mechanisms of chemotaxis MSC homing in fracture healing.

## Data Availability

The authors do not wish to share the data for the moment since further study is in progress.

## Abbreviations

MSC: Mesenchymal stem cell
HIF: Hypoxia-inducible factor
SDF-1: Stromal cell-derived factor
CXCR: C-X-C chemokine receptor
FAK: Focal adhesion kinase
SW: Short-Wave therapy treatment group
Con: Control group
MG: MSC labeled by the GFP
